# Double plating is associated with higher fixation strength than single plating in osteoporotic fractures of the scapular spine: a biomechanical study

**DOI:** 10.1007/s00402-021-03879-z

**Published:** 2021-04-12

**Authors:** J. Christoph Katthagen, Julia Sußiek, Andre Frank, Jens Wermers, Benedikt Schliemann, Michael J. Raschke

**Affiliations:** Department of Trauma-, Hand- and Reconstructive Surgery, University Hospital Muenster, Waldeyer Str. 1, 48149 Muenster, Germany

**Keywords:** Scapula spine, Double plate, Single plate, Fracture, Biomechanics, Reverse shoulder arthroplasty

## Abstract

**Introduction:**

The number of atraumatic stress fractures of the scapular spine associated with reverse shoulder arthroplasty is increasing. At present, there is no consensus regarding the optimal treatment strategy. Due to the already weakened bone, fractures of the scapular spine require a high fixation stability. Higher fixation strength may be achieved by double plating. The aim of this study was to evaluate the biomechanical principles of double plating in comparison to single plating for scapular spine fractures.

**Methods:**

In this study, eight pairs (*n* = 16) of human shoulders were randomised pairwise into two groups. After an osteotomy at the level of the spinoglenoid notch, one side of each pair received fracture fixation with a single 3.5 LCP (Locking Compression Plate) plate. The contralateral scapular spine was fixed with a 3.5 LCP and an additional 2.7 LCP plate in 90–90 configuration.

The biomechanical test protocol consisted of 700 cycles of dynamic loading and a load-to-failure test with a servohydraulic testing machine. Failure was defined as macroscopic catastrophic failure (screw cut-out, plate breakage). The focus was set on the results of specimens with osteoporotic bone quality.

**Results:**

In specimens with an osteoporotic bone mineral density (BMD; *n* = 12), the mean failure load was significantly higher for the double plate group compared to single plating (471 N vs. 328 N; *p* = 0.029). Analysis of all specimens (*n* = 16) including four specimens without osteoporotic BMD revealed no significant differences regarding stiffness and failure load (*p* > 0.05).

**Conclusion:**

Double plating may provide higher fixation strength in osteoporotic bone in comparison to a single plate alone. This finding is of particular relevance for fixation of scapular spine fractures following reverse shoulder arthroplasty.

**Level of evidence:**

Controlled laboratory study.

## Introduction

Fractures of the scapular spine are rare. However, the number of atraumatic stress fractures associated with reverse shoulder arthroplasty (RSA) is increasing, with rates of up to 4.3% reported in the literature [1–6]. The altered joint kinematics with RSA lead to higher tensile forces of the deltoid stressing the scapular spine, especially in patients who receive RSA for cuff tear arthropathy. This is supported by a current review by King et al. who found a significantly higher incidence of scapular spine fractures in patients who underwent RSA for massive cuff tears (3.8%), whereas patients with RSA for acute proximal humeral fracture sustained no such fractures [2]. Finally, a postero-superiorly directed screw used to fix the glenoid baseplate may also further decrease the stability of the scapular spine [7].

To date, there is controversy regarding the optimal treatment strategy for fractures of the scapular spine as both conservative and operative treatment can be considered [1, 3–6, 8]. Fixation of fractures associated with RSA was related to a high complication rate (non-unions, fixation failure), leading to a preference of non-surgical measures. However, the level of evidence is low. Nonetheless, fixation failure may occur due to insufficient stability of the fixation, particularly in osteoporotic bone. Double plating may be an option to increase fixation stability, which was recently shown for locations such as the proximal humerus or the proximal forearm [9–13].

The aim of the present study was to compare the biomechanical properties of double plating of the scapular spine in comparison to single plating with regard to stiffness and failure loads. It was hypothesized that double plating would provide higher fixation strength in both osteoporotic and non-osteoporotic bone.

## Materials and methods

Eight pairs (7 female, 1 male) of human fresh frozen shoulders (*n* = 16) were examined in this study. The average age was 86 ± 9 years. All of the donors provided written consent of their own free will for the use of their bodies for research purposes (IRB: 2014–421-f-N).

Prior to testing, all specimens underwent a CT scan to measure the bone mineral density (BMD) of the proximal humerus according to Krappinger et al. [14]. We assumed that, if the BMD of the proximal humerus is osteoporotic, the scapular spine from the same side would also have a reduced BMD. However, the BMD of each body side was measured separately. To the best of our knowledge, cut-off values for osteoporotic BMD of the scapular spine are not yet available in the literature.

The specimens were pairwise randomised into two groups. One side of each pair received fracture fixation with a 3.5 LCP plate (SP = single plate). The contralateral specimen was fixed with a 3.5 LCP plate and an additional 2.7 LCP in 90–90 configuration (DP = double plate).

### Specimen preparation

All soft tissue except the deltoid muscle was removed. The deltoid was then dissected approximately 2 cm from its scapular origin. The medial aspect of the scapula was embedded with RenCast polyurethane (Gößl + Pfaff, Karlskron, Germany) in a custom-made rectangular frame. To generate a reproducible setting, the distance between the glenoid cavity and the frame measured 7 cm in all specimens.

**Fig. 1:**
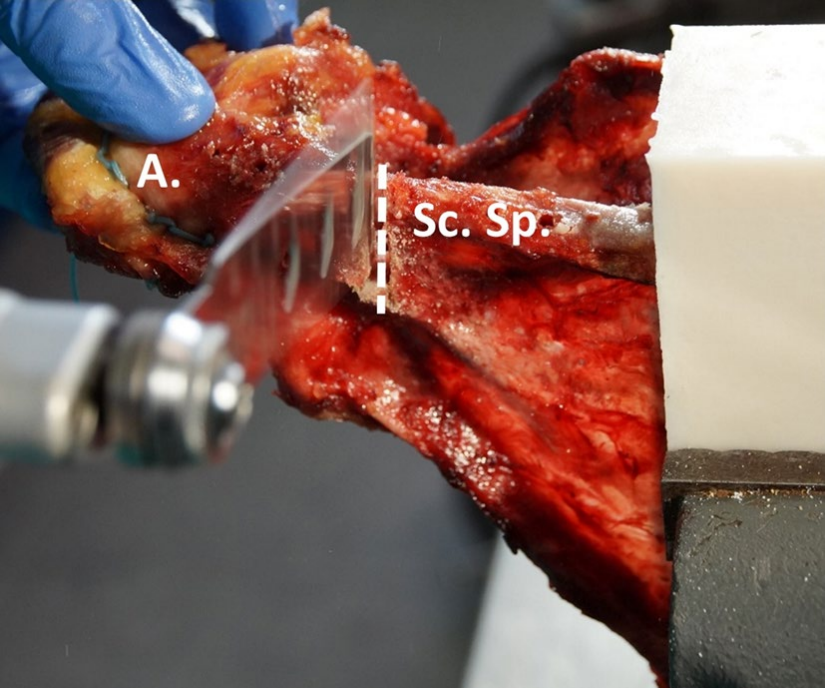
Osteotomy at the level of the spinoglenoid notch (*A* acromion, *Sc.Sp* scapular spine)

Each specimen received a 3.5 6-hole LCP plate (DePuy Synthes, Umkirch, Germany) on the dorsal aspect of the scapular spine. The plate was precontoured with bending tongs to the individual anatomical shape of the scapular spine. Afterwards the middle of the plate was placed above the spinoglenoid notch. With two 3.5 cortical screws (DePuy Synthes, Umkirch, Germany) placed in the penultimate screw holes of each side, the proper plate position was secured. In the remaining four holes, 3.5 mm locking screws (DePuy Synthes, Umkirch, Germany) were inserted. All screw holes were predrilled before and a length gauge was used to determine the appropriate screw length. To tighten the locking screws, a torque limiter was used. Once all screws were inserted, they were again removed prior to the osteotomy. A standardized fracture of the scapular spine was created with an oscillating saw. The fracture gap was set at the level of the spinoglenoid notch corresponding to a type III acromion fracture according to Crosby et al. (Fig. 1). [15]

**Fig. 2:**
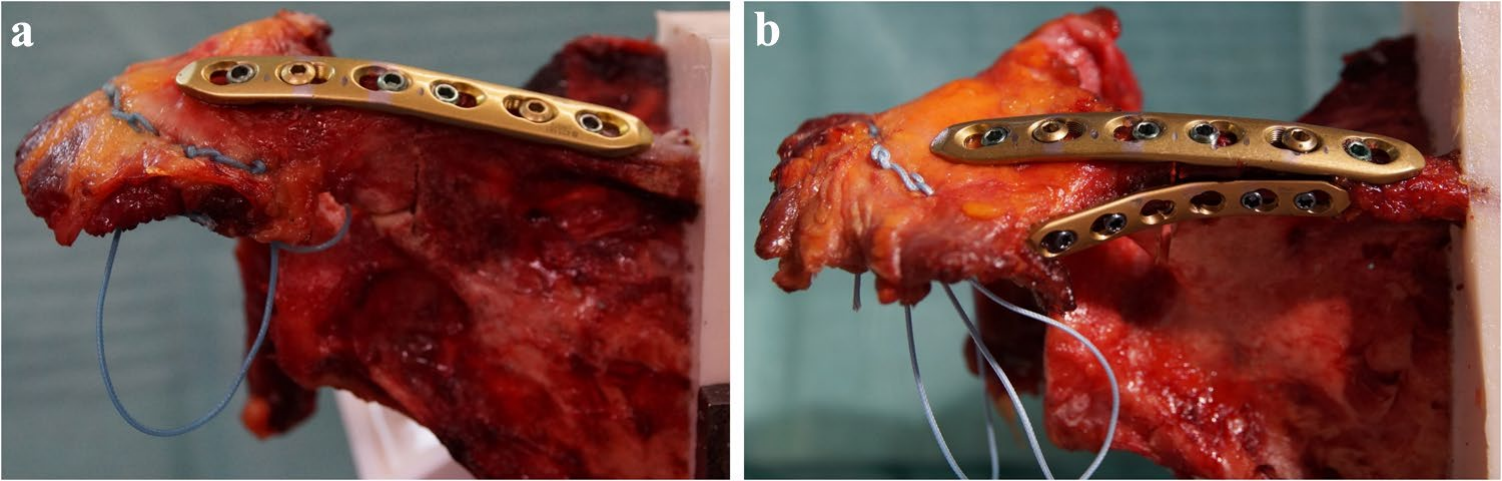
Plate configuration for the SP (**a**) and DP osteosynthesis (**b**)

After performing the osteotomy, the 3.5 mm LCP plate was refixed as described above. In the specimens of the DPO group, an additional 2.7 mm 6-hole LCP plate (DePuy Synthes, Umkirch, Germany) was mounted from inferior to the scapular spine (Fig. 2). After precontouring, the plate was fixed with four 2.7 mm locking screws. Again, all screw holes were predrilled before and a length gauge was used to determine the appropriate screw length.

### Biomechanical test setup

**Fig. 3:**
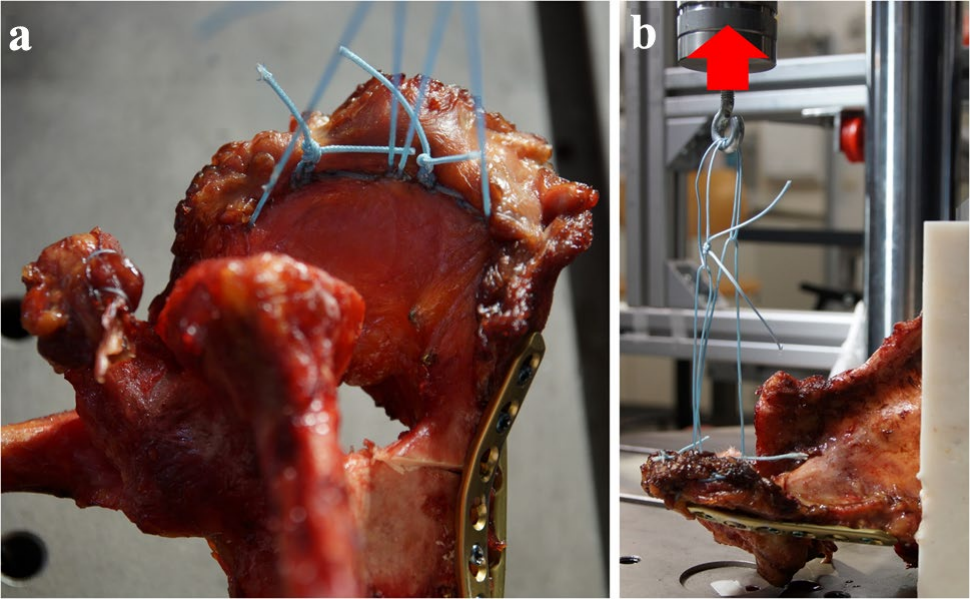
Fixation of the suture wires for load transmission to the deltoid muscle (**a**), test setup (**b**) the red arrow indicates the direction of loading

For biomechanical testing, the specimens were mounted on the baseplate of a servohydraulic testing machine (Instron 8874, Pfungstadt, Germany) in alignment with the load transmitter. According to the test setup by Spiegl et al. a wire system was chosen for load transmission [16]. Two suture wires (US 5, FiberWire, Arthrex GmbH, Munich, Germany) were attached to the medial and posterior aspects of the deltoid muscle. The wires were fixed with a locking stitch technique as close as possible to the bony insertion (Fig. 3a).

Each suture wire was knotted into a loop. A third loop was placed through the two others and connected to the eyelet of the loading cell. This additional loop acted as a load transmitter, ensuring a homogeneous and reproducible load distribution to the deltoid muscle (Fig. 3b).

The biomechanical test protocol consisted of a preload of 30 N [17] followed by dynamic testing with 700 cycles covering a minimum load of 30 N and a maximum load of 130 N. The first 200 cycles were used as preconditioning; therefore, results will be reported from cycle 200 onward. The maximum cyclic load based on the average muscle force of the medial part of the deltoid muscle measured by Ackland et al. for an abduction movement with a reverse prosthesis (112 N), the predicted values by Karlsson and Peterson for an abduction movement (60° with 1 kg–130 N) [18, 19] and the physiological cross sectional area defined by Veeger et al. [20]. After cyclic loading, a position/displacement-controlled load-to-failure test with a displacement rate of 60 mm/min in accordance to the previous established test protocol by Spiegl et al. was performed [16]. Failure was defined as catastrophic failure (screw cut-out, plate breakage).

### Data analysis

A customized Matlab script (Matlab 2019b, Massachusetts, USA) was used to calculate the construct stiffness as well as motion at the fracture site measured via the displacement of the actuator during the cyclic loading and the load-to-failure test.

GraphPad Prism (GraphPad 8.3.1, San Diego, USA) was used for the analysis of the motion at the fracture site and construct stiffness. First of all, the stiffness was analysed only for the 12 osteoporotic scapulae with an unpaired t test. For the entire group a normal distribution was not given, thus the Wilcoxon signed rank test was used for the statistical comparison.

For the load-to-failure analysis the Shapiro–Wilk test revealed a normal distribution. Statistical analysis was performed in the first step only for the osteoporotic scapulae with an unpaired t-test. In a second step, the load to failure was analysed for the entire group with a paired t-test. The level of significance was set *α* = 0.05.

## Results

Bone mineral density was osteoporotic (threshold < 90 mg/cm^3^) in 12 (mean BMD of the proximal humerus non-osteoporotic: 119.2 mg/cm^3^ mean BMD osteoporotic: 61.8 mg/cm^3^) scapulae.

**Fig. 4:**
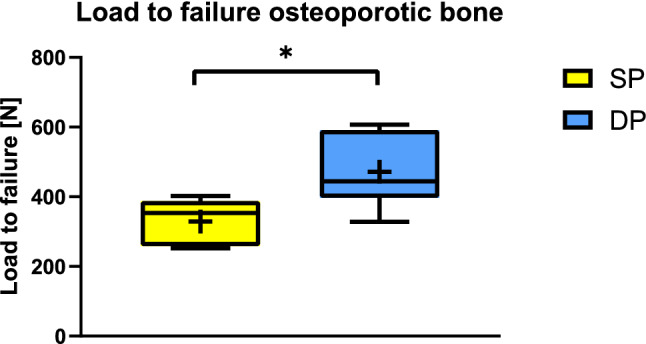
Failure loads after DP compared to SP for specimens with osteoporotic bone (*SP* single plate, *DP* double plate)

The construct stiffness during cyclic loading was similar with single and double plating for osteoporotic scapular spine fractures (SP 47.31 N/mm, SD ± 7.7; DP 44.16 N/mm, SD ± 7.4; *p* = 0.507). However, the mean load to failure of specimens with osteoporotic BMD was significantly higher with double plating compared to single plate fixation (471 N, SD ± 131 vs. 328 N, SD ± 60; *p* = 0.029; Fig. 4).

**Fig. 5:**
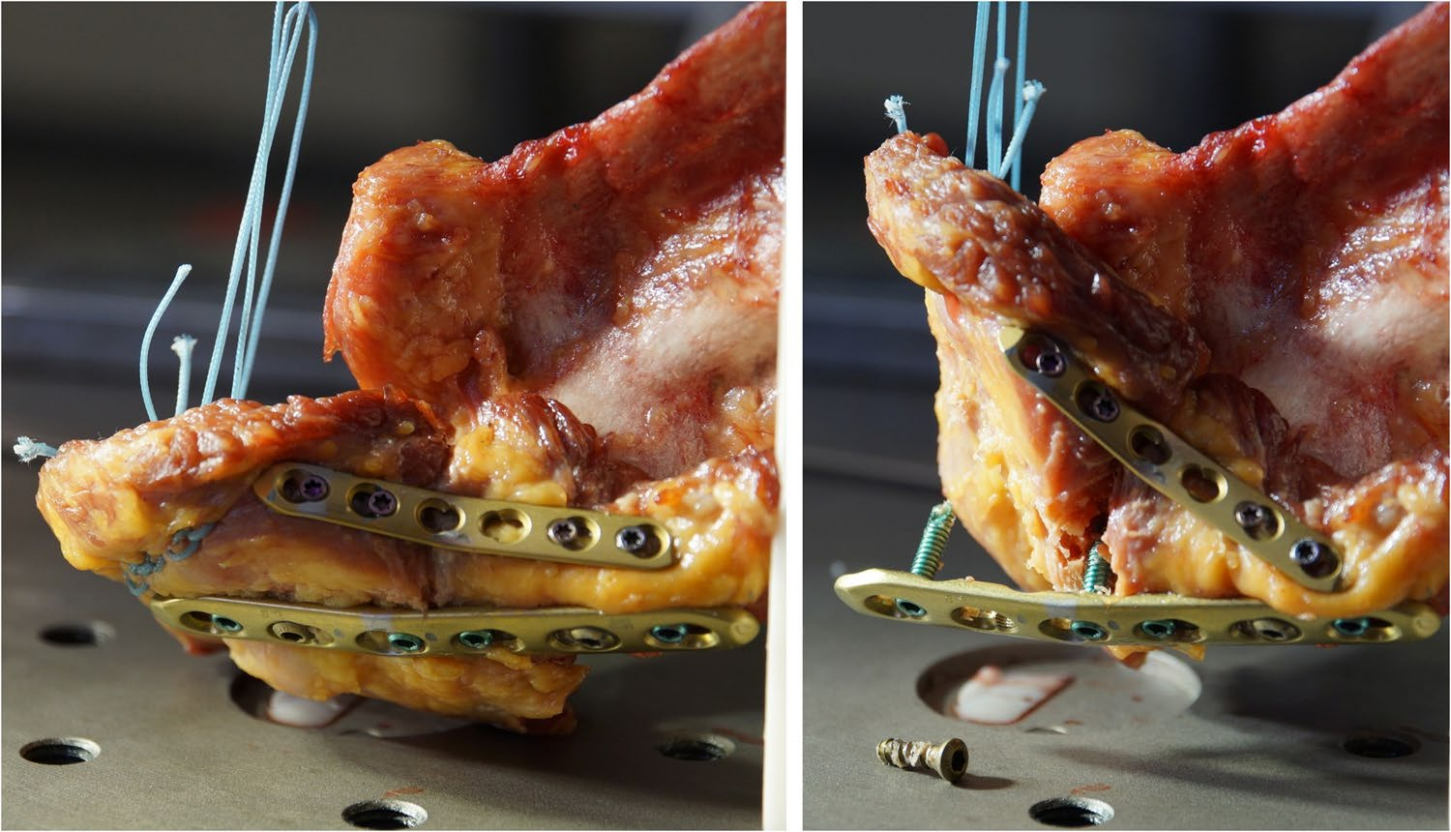
Example of the catastrophic load to failure with plate and screw cut out

Analysis of the results of all specimens (*n* = 16) including four specimens without osteoporotic BMD revealed no significant differences regarding stiffness (SP 53.46 N/mm, SD ± 21.6; DP 46.19 N/mm, SD ± 8.62; *p* = 0.375) and failure load (SP 356 N, SD ± 69, DP 451 N, SD ± 101, *p* = 0.074). The motion at the fracture site during cyclic loading was similar in both groups (*p* = 0.86). The mode of failure was a screw cut-out in all but two specimens in both groups (Fig. 5). In the remaining specimens (DP group), a fracture of the acromion or the spine medial to the plate was the mode of failure.

## Discussion

The most important finding of the present study is that double plating of scapular spine fractures provides significantly higher failure loads when compared to single plating in specimens with osteoporotic bone quality.

As the number of reverse shoulder arthroplasty is increasing, a rising number of complications, including fractures of the scapular spine must be expected. If fractures are non-displaced, conservative treatment may be successful [3, 6]. However, a displacement at the fracture site comes along with secondary impingement due to the inferior tilt of the distal fragment and decreased strength of the deltoid muscle. Dependent on the exact location of the fracture, both the middle and posterior parts of the deltoid can be involved. Simplified, the more medial the fracture is located, the more severe is the functional impairment. Particularly after RSA the function and mobility of the arm relies on proper deltoid function. Displaced fractures lead to even worse functional outcomes if not treated properly. However, there is no consensus in the literature on how to treat these fractures in elderly with RSA [21].

Currently, we see an indication for surgical therapy in patients with dislocation of more than 5 mm, with resulting angulation of the acromion causing delta insufficiency and in patients with persisting symptoms such as pain and dysfunction as well as in symptomatic non-unions.

There are only a few biomechanical studies on plate fixation of scapular spine fractures in the literature.

In a study of 19 scapula sawbones, Kicinski et al. compared the biomechanical properties of three different types of plates (lateral clavicular plate, a locking compression plate, and a reconstruction plate) and found the highest failure loads for the locking compression plate (506 N) [17]. As in the actual study the fracture was created at the level of the spinoglenoid notch (type III fracture), but the load was directly transmitted on the acromion.

More recently, Ting et al. could demonstrate that subcutaneous border plating is biomechanically superior to plating in the supraspinatus fossa [22]. Both studies evaluated measures for additional fracture fixation. With a lateral hook plate, Ting et al. presented the possibility to reduce the failure of the lateral acromion. Similar to the hook plate, in the actual study, the additional plate was chosen to provide a second strut and to improve stability to single border plating alone, which may be of advantage in osteoporotic bones as usually found in patients with RSA.

For the biomechanical testing, Ting et al. and Kicinski et al. chose pointwise loading of the acromion (pressure applied from superior), while in this study the direct loading of the deltoid allowed a more physiological load-transmission. Therefore, a direct comparison of the load to failure loads is not applicable.

Clinically, Schoch et al. reported functional results of both conservative and operative treatment of fractures of the acromion and the scapular spine in patients with RSA [23]. They observed an incidence of 2.1% with 12 fractures (of which 11 were scapular spine fractures) in 583 RSA implantations. Of these 12 fractures, the first six were treated with plate fixation, the latter six remained conservative. After a mean follow-up of 4.5 years, the mean Constant score in the operative group was 55.4 points compared to 55.3 points in the conservative group. However, a subgroup analysis revealed that surgically treated patients had improved function whereas those who underwent conservative treatment benefitted mostly from pain relief.

Overall, only case reports with small numbers of patients with scapular spine fractures can be found in the literature [5, 8, 24]. Debeer and Robyns reported excellent pain relief and restoration of the pre-fracture function in a patient with a fracture of the scapular spine after RSA [8]. Toft and Moro presented a series of 5 patients with a RSA associated scapular spine fracture that underwent double plate fixation with a 5-hole LCP and a 5–6 hole quarter tubular plate [5]. At the follow-up, functional scores were similar to those prior to the fractures. The authors concluded that operative fixation is a valuable option for these patients.

From a biomechanical point of view, fixation of scapular spine fractures must be strong enough to withstand the deltoid force to restore shoulder function and has to maintain stability until bone union is achieved. It is widely known that fixation strength depends on the bone quality. In the last decade, many efforts have been made to improve fracture fixation strength in patients with osteoporotic bone structure. Apart from locked plating, which can be regarded as a standard procedure in osteoporotic fractures, additional measures such as augmentation with bone cement or allografts are nowadays widely used [25, 26]. Recently, double plating has come into focus of both clinical and biomechanical research as it seems to improve fixation strength, i.e. in proximal humerus and olecranon fractures, and it is also used in scapular spine fractures [9, 11–13, 23]. The present study proofs for the first time that double plating is associated with higher fixation strength than single plating in osteoporotic fractures of the scapular spine. Given the increasing number of reverse shoulder arthroplasty, this might be of clinical relevance in the future, when the number of scapular spine fractures associated with RSA equally increases.

For the clinical application of double plating at the scapular spine, several aspects must be considered. For the second plate, no additional approach is needed. As the 2.7 LCP is applied on the origin of the posterior part of the deltoid, in clinical routine no or just very little detachment is performed. However, clinical case series are needed to rule out potential insufficiency of the posterior deltoid.

Some limitations apply to the present study. First of all, being a time zero biomechanical test set-up only the time zero-testing could be evaluated and the in-vivo healing could not be considered. The number of specimens is low (*n* = 16, 8 pairs). However, with the majority being female and with an average age of 86 years, it represents the typical patient who sustains a scapular spine fracture associated with RSA in the clinical setting. In addition, only unidirectional loading was performed. However, this refers to a worst-case scenario, simulating a maximum pull of the deltoid muscle, which again closely imitates a RSA situation.

## Conclusion

Double plating may provide higher fixation strength in osteoporotic bone in comparison to a single plate alone. This finding is of particular relevance for fracture fixation following shoulder arthroplasty.

## Data Availability

Not applicable.
